# Supplementary material for: Guided morphogenesis through optogenetic activation of Rho signalling during early Drosophila embryogenesis
Source: Nat Commun. 2018 Jun 18;9:2366. doi: 10.1038/s41467-018-04754-z (PMC6006163; doi:10.1038/s41467-018-04754-z)
Supplement: Supplementary file 1 — Supplementary Information [file 41467_2018_4754_MOESM1_ESM.pdf]

Guided morphogenesis through optogenetic activation of Rho signaling during early *Drosophila* embryogenesis

Izquierdo et al.

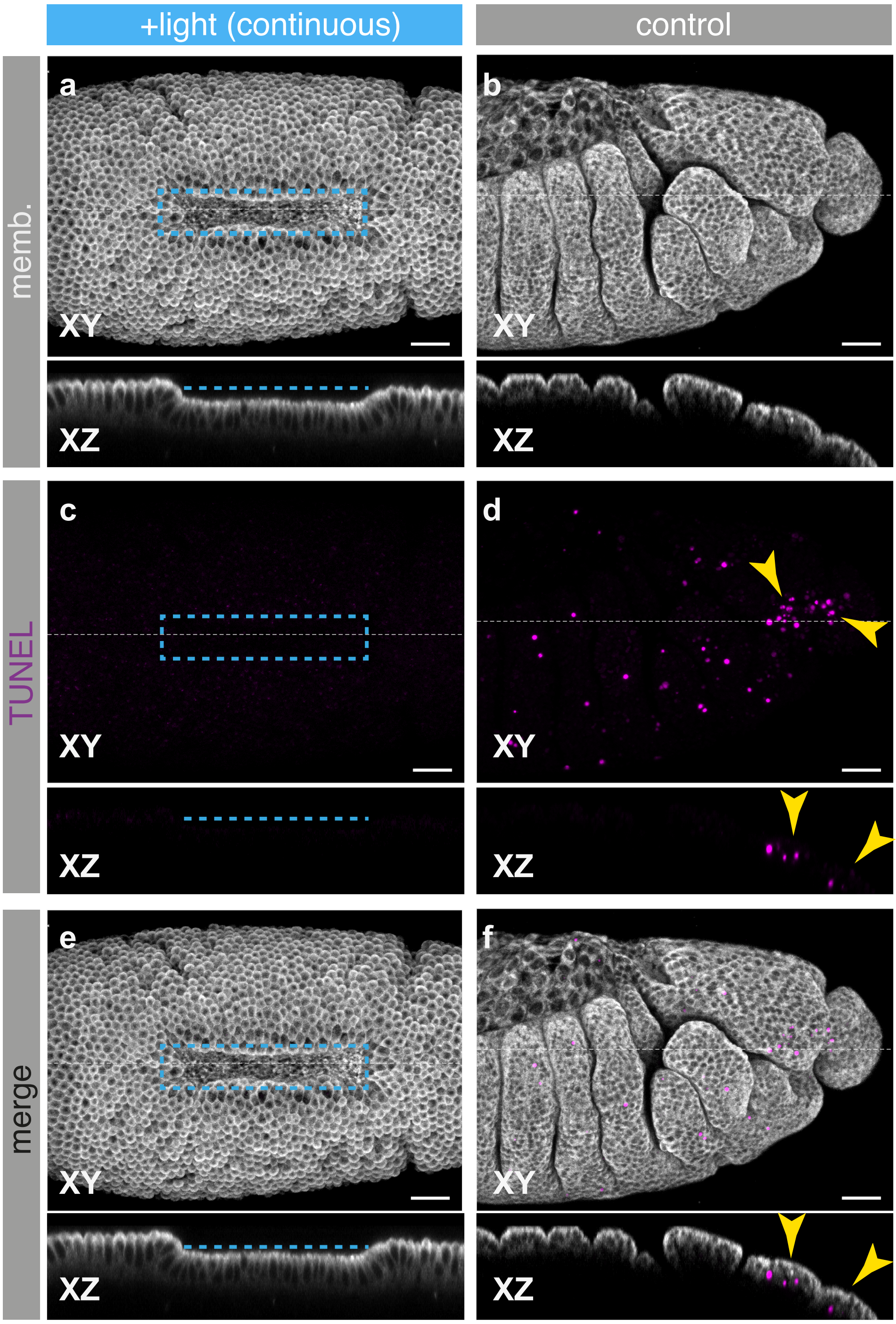

Supplementary Figure 1: Optogenetic activation and apoptosis
(a-f) Confocal images of representative embryos (N=5) co-expressing CIBN::pmGFP and RhoGEF2-CRY2::mCherry double-labeled with TUNEL and antibody staining. Embryos were pooled together, dechorinated, fixed and stained with anti-GFP to label the plasma membrane and fluorescein-dUTP to label apoptotic cells. All panels show both top and crossectional views of the embryos. **(a,c,e)** A gastrulating embryo (stage 5) was locally photo-activated to induce an invagination. The blue dotted rectangle indicates the site of photo-activation. **(a)** Anti-GFP staining labeling the cell membranes. **(c)** TUNEL staining does not detect any apoptotic cells. **(e)** Merge of membrane and TUNEL labeling. **(b,d,f)** A control non photo-activated stage 14 embryo, yellow arrowheads indicate positively labeled apoptotic cells. **(b)** Anti-GFP stain labeling the cell membranes. **(d)** TUNEL staining identifies several apoptotic cells. **(f)** Merge of membrane and TUNEL staining. Scale bars, 20 μm.

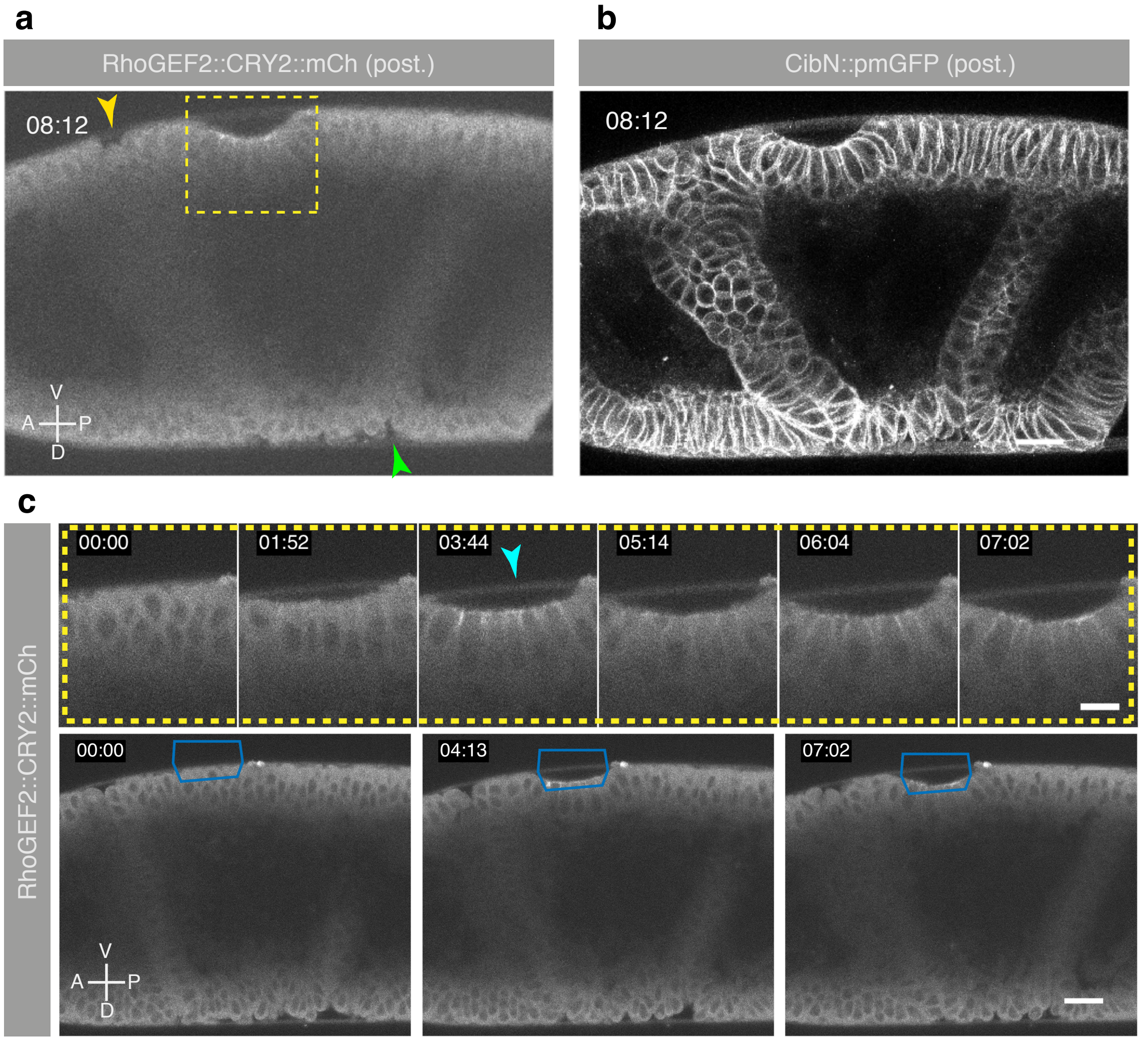

Supplementary Figure 2: Induction of an ectopic invagination in the late gastrulating embryo

(a-c) A representative embryo (N=4) of stage 7-8 co-expressing CIBN::pmGFP and RhoGEF2-CRY2::mCherry. A region encompassing ~7 cells in the ventral side of the embryo was photo-activated in an optical cross-section for a total of 5μm from the apical-most plane (blue box in (c)). Sustained photo-activation was alternated with mCherry excitation, the protocol lasted for ~8 min. after which both mCherry and GFP signals were recorded for the whole embryo (a-b). **(a)** Confocal still frame showing RhoGEF2-CRY2::m-Cherry after photo-activation. Arrowheads indicate embryonic stage 8 morphogenetic land-marks, in yellow the cephalic furrow, in green the posterior dorsal fold. Detailed time-course recordings of the region enclosed the dashed yellow box are showed in (c). **(b)** Confocal still frame showing membrane outlines (CIBN::pmGFP) after photo-activation. Scale bar is 20μm. **(c)** Confocal still frames from a time-lapse recording of the embryo in (a-b) for which a detailed view (top) and a view of the whole embryo are shown. Arrowhead in cyan indicat-ed the vitilline membrane. The blue box indicates the area of photo-activation. Scale bars are 10μm (top) and 20μm (bottom).
