# Supplementary material for: Guided morphogenesis through optogenetic activation of Rho signalling during early Drosophila embryogenesis
Source: Nat Commun. 2018 Jun 18;9:2366. doi: 10.1038/s41467-018-04754-z (PMC6006163; doi:10.1038/s41467-018-04754-z)
Supplement: Supplementary file 3 — Description of Additional Supplementary Files [file 41467_2018_4754_MOESM3_ESM.docx]

**Description of Additional Supplementary Files**

File Name: Supplementary Movie 1

Description: RhoGEF2 plasma membrane recruitment causes tissue-level responses matching the geometrical pattern of photo-activation. Confocal time-lapse recordings of three representative embryos co-expressing RhoGEF2-CRY2::mCherry (top) and CIBN::pmGFP (bottom). Each recording shows integrated intensity projections of 3µm within the dorsal epithelium. Top: RhoGEF2-CRY2::mCherry localizes to the cytoplasm which upon photo-activation is recruited to the plasma membrane. Bottom: the plasma membrane localization of CIBN::pmGFP during photoactivation. Dashed lines correspond to the outline of the embryo. For each embryo, photo-activation with two-photon illumination was restricted to a geometrical pattern, left to right: circle, triangle and square. Blue dot indicates the occurrence of two-photon illumination (top-left corner). Scale bars are 10 μm.

File Name: Supplementary Movie 2

Description: RhoGEF2 optogenetic activation causes apical constriction and tissue invagination. Confocal time-lapse recording of the dorsal epithelium of a representative embryo co-expressing CIBN::pmGFP (not shown), RhoGEF2-CRY2::mCherry (not visible) and a membrane marker Gap43::mCherry (white). Photo-activation with two-photon illumination occurred within a rectangular region (initial dashed rectangle). Top: photoactivated cells appear marked with a blue dot. As the cells constricted and the epithelium folded inwards, their apices were followed by adjusting the plane of acquisition (on the top-right corner notice the annotation for changes in zplane). Bottom: Initial plane of acquisition where the apices of non-activated cells remained. Cells immediately adjacent to the area of activation are marked with a magenta dot, and the remaining non-activated cells are marked with a gray dot. Blue dot indicates the occurrence of two-photon illumination (top-left corner). Scale bars are 10 μm.

File Name: Supplementary Movie 3

Description: Recruitment of RhoGEF2 at the plasma membrane causes myosin II apical enrichment and tissue contraction. Confocal time-lapse recording of a representative embryo co-expressing CIBN::pmGFP, RhoGEF2-CRY2 and the myosin II regulatory light chain reporter Sqh::mCherry. The recording shows integrated intensity projections of 3µm of the dorsal epithelium. Continuous photo-activation with two-photon illumination was restricted to a rectangular area (dashed line). Top: myosin II signal (white) starts to accumulate in the apices of the cells within two minutes of photo-activation. Bottom: A merge of myosin II signal (red) and the outline of the cells as reveled by the membrane-bound CIBN::pmGFP (green). Upon myosin II accumulation, cells responded by contracting their apices. Blue dot indicates the occurrence of two-photon illumination (top-left corner). Scale bars are 10 μm.

File Name: Supplementary Movie 4

Description: Induction of pulsatile contractions. Confocal time-lapse recording of the dorsal epithelium of representative embryos coexpressing CIBN::pmGFP, RhoGEF2-CRY2::mCherry (not visible) and the plasma membrane marker Gap43::mCherry (white). For the three embryos, the dorsal epithelium was subjected to three different illumination protocols presented in the following order: discontinuous, continuous, and single pulse of activation. The dashed rectangle indicates the area of photo-activation. The blue dot indicates the time of photo-activation. Scale bars, 10 μm.

File Name: Supplementary Movie 5

Description: Pulsatile contractions and myosin II dynamics. Confocal time-lapse recording of a representative embryo co-expressing CIBN::pmGFP, RhoGEF2-CRY2 and the myosin II regulatory light chain reporter Sqh::mCherry. The recording shows integrated intensity projections within 5µm of the cells’ apical-most plane. The dorsal epithelium was subjected to a strong initial two-photon illumination pulse followed by a continuous lower two-photon excitation for image acquisition. Presented in the video, is a merge of myosin II signal (red) and CIBN::pmGFP (white) at the membrane. Blue rectangle indicates the occurrence of two-photon illumination within the dashed rectangle. Scale bars are 10 μm.

File Name: Supplementary Movie 6

Description: Relationship between the geometrical pattern of optogenetic activation and orientation of contractile behavior. Confocal time-lapse recordings of the dorsal epithelium of six representative embryos co-expressing CIBN::pmGFP (not shown), RhoGEF2-CRY2::mCherry (not visible) and the plasma membrane marker Gap43::mCherry. For the different embryos, photo-activation occurred within a rectangular region with a varied width to height ratio, here presented sequentially in the following order: 3:1, 1:1, 2:1, 4:1, 6:1, 9:1. Dashed line indicates the area of photo-activation with two-photon illumination. Blue dot indicates the occurrence of two-photon illumination (top-left corner). Scale bars are 10 μm.
